# Supplementary figures and images for: Extracellular Vesicles Isolation from Large Volume Samples Using a Polydimethylsiloxane-Free Microfluidic Device
Source: Int J Mol Sci. 2023 Apr 27;24(9):7971. doi: 10.3390/ijms24097971 (PMC10178709; doi:10.3390/ijms24097971)

**Figure S1.** Isolated particle size distribution by isolation method per sample.

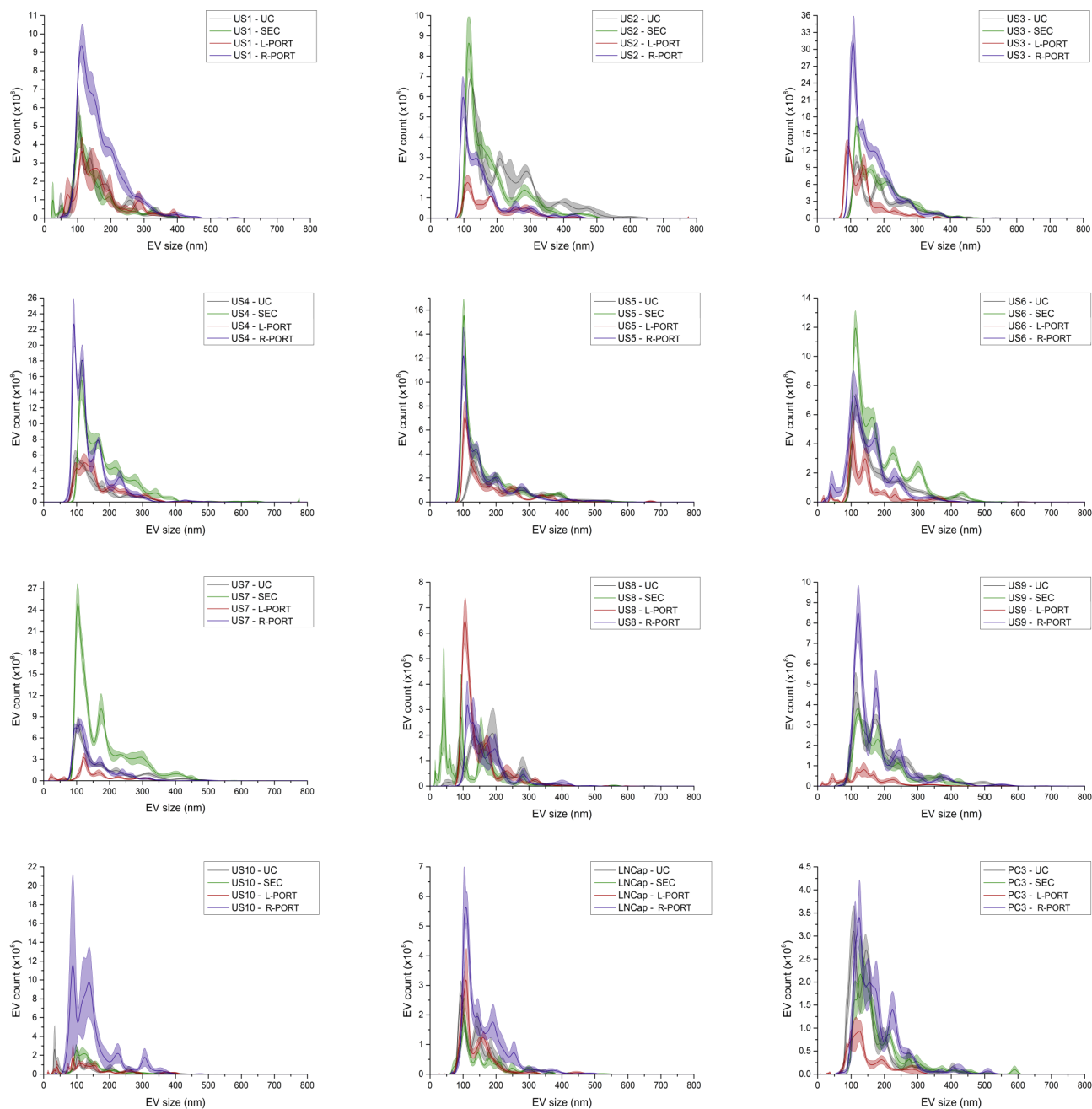

Supplement: Supplementary file 1 [file ijms-24-07971-s001.zip › ijms-2287119-supplementary.pdf]
